# Chidamide, a histone deacetylase inhibitor, inhibits autophagy and exhibits therapeutic implication in chronic lymphocytic leukemia

**DOI:** 10.18632/aging.103536

**Published:** 2020-08-27

**Authors:** Yi-Lin Kong, Bi-Hui Pan, Jin-Hua Liang, Hua-Yuan Zhu, Li Wang, Yi Xia, Jia-Zhu Wu, Lei Fan, Jian-Yong Li, Wei Xu

**Affiliations:** 1 Department of Hematology, The First Affiliated Hospital of Nanjing Medical University, Jiangsu Province Hospital, Nanjing 210029, China; 2 Key Laboratory of Hematology of Nanjing Medical University, Nanjing 210029, China; 3 Collaborative Innovation Center for Cancer Personalized Medicine, Nanjing 210029, China

**Keywords:** chronic lymphocytic leukemia, autophagy, chidamide, histone deacetylase inhibitor

## Abstract

Novel agents have made the management of chronic lymphocytic leukemia (CLL) more promising and personalized. However, long-term treatment is still warranted which may result in toxicity and resistance. Thus, new combination therapy may help achieve deeper remission and limited-duration therapy. Histone deacetylase inhibitors (HDACi) can affect many tumors by modulating key biological functions including autophagy. Studies have shown that some novel targeted agents including ibrutinib induce autophagy. This study aimed to explore the effect of oral HDAC inhibitor, chidamide, on CLL cells as well as the role of autophagy in this process. Here, we showed that autophagy flux in CLL cells was inhibited by chidamide via post-transcriptional modulation and chidamide had cytostatic and cytotoxic effects on CLL cells. Besides, the pro-survival role of autophagy in CLL cells was validated by using autophagy inhibitor and knocking down critical autophagy gene. Notably, a combination of chidamide and ibrutinib showed significant synergism and downregulated ibrutinib-induced autophagy. This work highlights the therapeutic potential of chidamide via its effect on autophagy, especially in combination with ibrutinib.

## INTRODUCTION

Chronic lymphocytic leukemia (CLL) remains the most common adult leukemia in the Western world. The average age at diagnosis is 70 years [1]. As the incidence increases with age, the prevalence of CLL may increase further due to the demographic changes. Besides, the proportion of younger patients with early-stage CLL seems to increase due to more frequent blood tests. CLL in its clinical manifestations, treatment response, and disease course are heterogeneous, which makes the treatment of CLL particularly challenging [2]. In just over a decade, the treatment of CLL has made great progress, mainly in the development of new targeted therapies such as ibrutinib, venetoclax, duvelisib, and idelalisib. Despite these advances, CLL remains an incurable disease for patients who do not undergo allogeneic stem cell transplantation [3]. Therefore, exploring management with combination or sequential therapy to receive deep remission, reduce toxicity, and improve the quality of life is undoubtedly a new challenge in the new drug era.

In recent years, histone deacetylase inhibitor (HDACi) has attracted more attention as low-toxic and effective anti-tumor drugs. Class I HDACs (HDAC1, 2, 3, and 8) and HDAC10 are found to have relevance in cell survival and proliferation [4]. In this regard, chidamide seems promising in cancer therapy as an orally available, isotype-selective benzamide that targets HDACs 1, 2, 3, and 10, currently approved for patients with relapsed or refractory peripheral T-cell lymphoma (PTCL) who have received at least one systemic chemotherapy in China [5]. Also, it has shown a cytostatic effect and cytotoxicity in various cancers *in vitro* [6]. One recent report of a clinical trial of breast cancer also showed promising outcomes [7]. Beyond their roles in histone acetylation, HDACs control several key biological functions [8], and it has reported that HDAC inhibitors can achieve anti-tumor effects by regulating autophagy pathways [9–11].

Macroautophagy (hereafter referred to as autophagy) is an evolutionarily ancient and highly conserved catabolic process that promotes both cell survival and cell death in a context-dependent manner. It involves the progressive sequestration of cytoplasmic material by double-membraned organelles (known as autophagosomes) that ultimately fuse with lysosomes to initiate the degradation of their cargo [12]. In the context of cancer, the role of macroautophagy is complex, depending on the stage, type, and the driving oncogenes. So far, it is widely believed that autophagy prevents the development of cancer. Conversely, once cancer is established, some of the mechanisms whereby autophagic responses preserve the homeostasis of healthy tissues are hijacked by progressing cancers. Furthermore, protective autophagy activated by treatment-induced stress may be one of the mechanisms of drug resistance. The role of autophagy remains ambiguous in hematopoietic cancers. Our previous study found that compared with healthy controls, the expression level of autophagy-related genes in primary CLL cells was significantly increased [13]. Consistently, several studies found that abnormal levels of autophagy have important significance in the occurrence, development, and drug resistance of leukemia, moreover, inhibition of autophagy can induce cytotoxicity or enhance the effect of other drugs [14–18].

Based on previous studies demonstrating the aberration of autophagy in CLL, promising anti-tumor effect of chidamide and the tumor intervention based on autophagy regulation by HDAC inhibitors. We investigated the role of chidamide in CLL and explored its relation to autophagy modulation.

## RESULTS

### Chidamide decreased the autophagic flux in CLL cells

**Figure 1:**
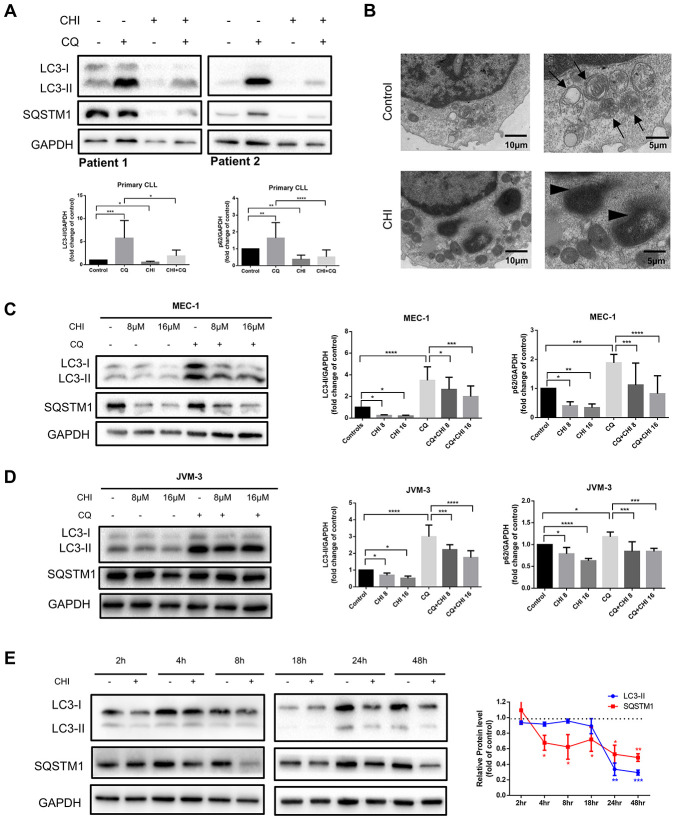
**Chidamide decreases autophagic flux in CLL cells.** (**A**) Immunoblotting analysis of autophagic flux in primary CLL cells after chidamide (CHI, 4μmol/L) treatment for 24 hours in the presence or absence of chloroquine (CQ, 10μmol/L). LC3 and SQSTM1 are indicated, as well as GAPDH that was used as a loading control. Shown are two representative blots from the samples of 16 patients. (**B**) Electron microscopic analysis of primary CLL cells in the presence or absence of chidamide (CHI, 4μmol/L) treatment for 24 hours. Arrows indicate autophagic structures and arrowheads indicate fragments of nucleus. (**C**, **D**) Immunoblotting analysis of autophagic flux in MEC-1 and JVM-3 cell lines respectively after chidamide (CHI, 8 or 16μmol/L) treatment for 24 hours in the presence or absence of chloroquine (CQ, 10μmol/L). (**E**) Levels of LC3 and SQSTM1 were assessed by immunoblotting in the presence or absence of chidamide (8μmol/L) in time-course experiments in MEC-1 cell line. GAPDH was used as a loading control. The bar graphs and the line graph showed the expression level of proteins with respect to the control groups. *P < 0.05; **P < 0.01; ***P < 0.001; ***P<0.0001.

To investigate the effect of chidamide on autophagy in CLL cells, we first analyzed the expression of LC3-II and SQSTM1/p62 protein, markers required for early and late steps of autophagy respectively. Since LC3-I may be less sensitive to detection by anti-LC3 antibodies and more labile than LC3-II [12], levels of LC3-II were compared with GAPDH rather than LC3-I. CLL cells from patients and two cell lines, MEC-1 and JVM-3, were incubated with chidamide for 24 hours. Decreased expression of LC3-II and SQSTM1 were found in both primary CLL cells (Figure 1A) and cell lines (Figure 1C, 1D). The observed difference may be caused by individual variation among patients. At the same time, autophagic structures were observed by transmission electron microscopy and fewer autophagosomes were seen after incubation of chidamide in primary CLL cells (Figure 1B). These results indicate chidamide decreased the number of autophagosomes in CLL cells.

As autophagic flux is a highly dynamic, multi-step process, reduced expression of LC3-II and SQSTM1 may indicate either an induction or an inhibition in the autophagic flux. Thus we used chloroquine, which can neutralize the lysosomal pH, to prevent lysosomal degradation [12]. LC3-II levels were lower in chidamide-treated cells than in untreated cells, even in the presence of chloroquine (Figure 1A, 1C, 1D), suggesting that chidamide may decrease the autophagic flux by inhibiting the formation of autophagosomes at upstream steps rather than inducing more LC3 turnover. However, a decrease of LC3-II was not accompanied by the accumulation of SQSTM1, an index of autophagic degradation [19]. To further observe the process, we performed a time-course analysis of LC3-II and SQSTM1 (Figure 1E). Interestingly, SQSTM1 significantly decreased several hours before the drop in LC3-II, and such a decrease was also seen in the presence of chloroquine, indicating that it may be due to an autophagy-independent mechanism.

Taken together, these data elucidate that chidamide decreases autophagic flux in CLL cells, which may be caused by obstructing the formation of autophagosomes or inducing autophagy-independent way of degradation.

### Chidamide inhibits autophagy by post-transcriptional regulation

### Chidamide regulates autophagy in CLL cells by PI3K/AKT/mTOR - independent mechanism

**Figure 2:**
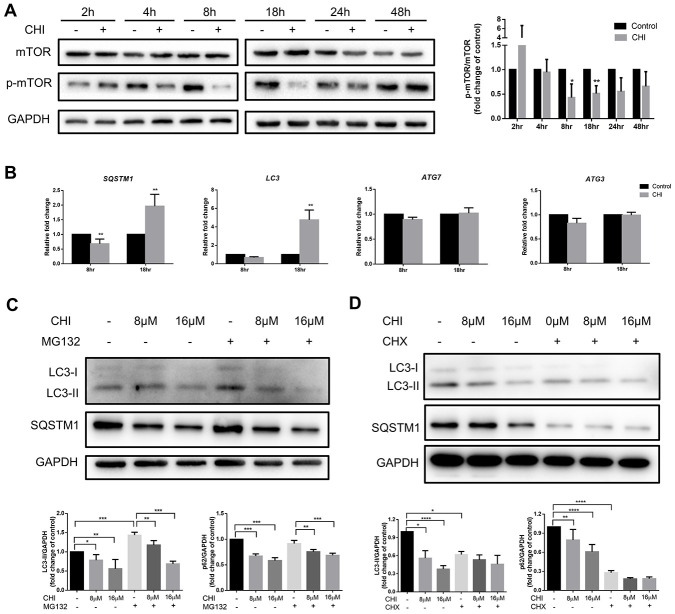
**Chidamide inhibits autophagy in mTOR-independent way by post-transcriptionally regulation in CLL cells.** (**A**) The effect of chidamide on the PI3K/AKT/mTOR pathway was assessed in MEC-1 cells by immunoblotting through the analysis of the phosphorylation status of mTOR during time course in presence or absence of chidamide (CHI, 16μmol/L). The bar graph showed the relative expression level of p-mTOR/mTOR with respect to the control groups. (**B**) The effect of chidamide on modulating autophagy gene expression in transcriptional level. MEC-1 cells were treated with chidamide (CHI, 16μmol/L) for 8 or 18 hours, then autophagy-related genes *LC3*, *SQSTM1*, *ATG7*, and *ATG3* were determined by qRT-PCR. ΔCt was used to measure statistical significance and the results were shown in the bar graphs. (**C**, **D**) MEC-1 cells were treated with chidamide (CHI, 8 or 16μmol/L) alone or in presence of MG132 (20μmol/L) or cycloheximide (CHX, 20μmol/L), collected after 24 hours and immunoblotted with antibodies against LC3 and SQSTM1. The bar graphs showed the expression level of proteins with respect to the control groups.

To explore the molecular mechanism of chidamide-mediated autophagy inhibition in CLL cells, we first detected the PI3K/AKT/mTOR pathway, which is one of the major signaling cascades for autophagy regulation [20]. Phosphorylation of mTOR was observed to increase at early time point (between 2 to 4 hours) then to decrease from 8 hours after treatment of chidamide (Figure 2A). However, early activation of this pathway did not affecct autophagy in CLL cells, whereas prolonged incubation with chidamide induced autophagy inhibition (Figure 1E) when the PI3K/AKT/mTOR pathway was inhibited, an event that normally activates autophagy, indicating the existence of other mechanisms that impair autophagy.

### Chidamide acts at the protein level to affect autophagy rather than regulating transcription levels

Therefore, we next investigated whether the regulation of autophagy-related genes could explain the effects of chidamide on autophagy in CLL cells. After treating with chidamide for 8 or 18 hours, the autophagy-related genes *LC3, SQSTM1, ATG7,* and *ATG3* were determined by qRT-PCR. ATG3, a ubiquitin-conjugating enzyme (E2) analog and ATG7, a ubiquitin-activating (E1) enzyme homolog, are both involved in activating and conjugating LC3 to phosphatidylethanolamine (PE) to form LC3-II [21]. The data showed that after 8 hour-treatment, the expression of *SQSTM1* decreased significantly (Figure 2B), which was consistent with the change in protein level (Figure 1E). However, contrary to a decrease in protein expression level, prolonged incubation increased the expression of both *LC3* and *SQSTM1* significantly (Figure 2B). Besides, there were no changes of *ATG7* and *ATG3* expression (Figure 2B). These results delineate that chidamide-mediated change of LC3-II expression might be due to decreased protein synthesis or protein stability instead of transcriptional regulation. Change of SQSTM1 expression may be caused by different mechanisms in a time-dependent manner. Transcriptional regulation may play a role early after chidamide treatment, while after prolonged incubation, factors beyond the transcriptome may contribute to the decreased expression level of protein.

### Chidamide interferes with the synthesis of autophagy-related proteins

To clarify how chidamide reduces autophagy-related protein expression, firstly, we used Chloroquine and MG132, which inhibited autophagy and proteasome respectively, to detect these two protein degradation ways. As previously shown, chidamide reduced the levels of LC3-II and SQSTM1 even in the presence of chloroquine (Figure 1A, 1C, 1D). Similarly, the loss of LC3-II and SQSTM1 was not prevented by MG132 (Figure 2C). The above results ruled out the effects of these two degradation pathways on the decrease of autophagy-related proteins. By contrast, under the condition that cycloheximide (CHX) inhibited protein synthesis, chidamide did not further reduce the expression of LC3-II and SQSTM1 (Figure 2D), suggesting that chidamide may have a similar effect on inhibiting protein synthesis.

Altogether, these results suggest that chidamide-mediated autophagy inhibition results from the interference with synthesis of autophagy-related proteins.

### Chidamide has cytostatic and cytotoxic effects on CLL cells

**Figure 3:**
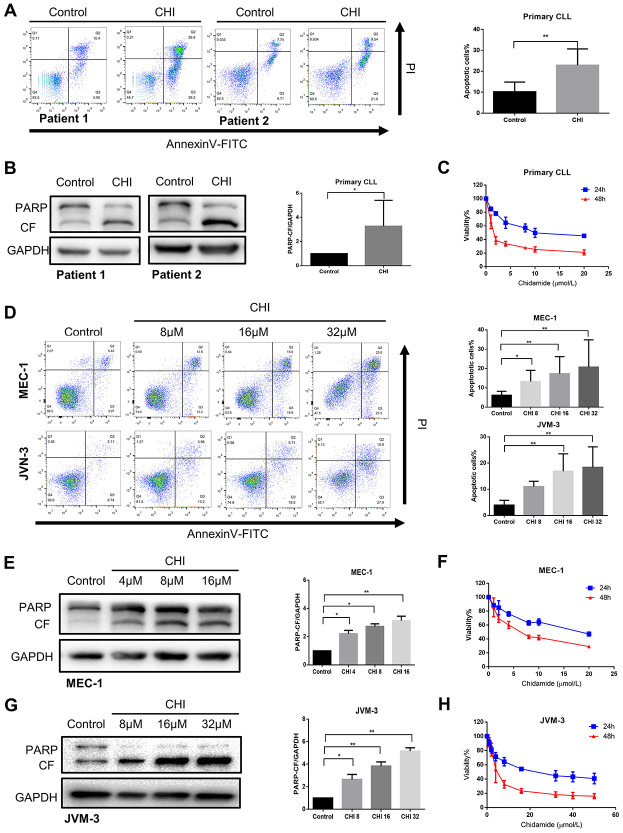
**Chidamide has cytostatic and cytotoxic effects on CLL cells.** (**A**) Flow cytometry using Annexin V–FITC/PI staining for cell apoptosis analysis. Primary CLL cells were incubated with 4μmol/L chidamide for 24 hours. Representative data shown are from 9 patients and three independent experiments of MEC-1, JVM-3 respectively. The bar graphs showed the percentage of apoptotic cells. (**B**) Immunoblotting analysis of poly (ADP-ribose) polymerase (PARP) in primary CLL cells after chidamide (CHI, 4μmol/L) treatment for 24 hours. Shown are 3 representative blots from the samples of 6 patients. The bar graph represents the relative PARP cleavage/GAPDH ratio measured by immunoblotting. (**C**) CCK8 assay for detecting metabolically active cells. Primary CLL cells were incubated with different concentrations of chidamide for 24 and 48 hours. Viability of cells compared with the corresponding controls was shown from three independent experiments respectively. (**D**) Cell apoptosis assay as described in (**A**). MEC-1, JVM-3 cell lines were incubated with indicated concentrations of chidamide for 24 and 48 hours. (**E**, **G**) Immunoblotting analysis of PARP as described in (**B**). MEC-1 and JVM-3 cell lines were incubated with indicated concentrations of chidamide for 24 hours. (**F**, **H**) CCK8 assay as described in (**C**). MEC-1, JVM-3 cell lines were incubated with different concentrations of chidamide for 24 and 48 hours.

Next, we sought to explore the biologic effects of chidamide on CLL cells. By performing flow cytometry with Annexin V/PI double staining, we found that the percentage of apoptotic cells increased after chidamide treatment in primary CLL cells ang cell lines (Figure 3A, 3D). Similar results were seen in immunoblotting analysis with increased cleavage of poly (ADP-ribose) polymerase (PARP) which mirrored an early indicator of apoptosis (Figure 3B, 3E, 3G). Growth curves detected by CCK8 assay revealed the sensitivity of primary CLL cells and cell lines to chidamide in a time- and dose-dependent manner (Figure 3C, 3F, 3H). Moreover, morphological observation by transmission electron microscopy showed more cells suffering from apoptosis with the characteristic of chromatin condensation and nuclear fragmentation after treatment of chidamide (Figure 1B). These results suggest that chidamide inhibits proliferation and induces apoptosis in CLL cells.

### Inhibition of autophagy recapitulates the effect of chidamide on CLL cells

**Figure 4:**
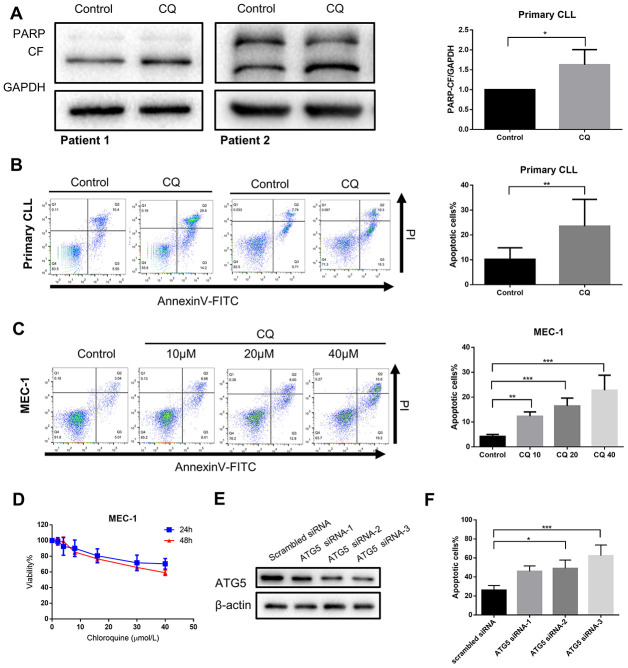
**Inhibition of autophagy recapitulates the effect of chidamide.** (**A**) Immunoblotting analysis of poly (ADP-ribose) polymerase (PARP) in primary CLL cells after chloroquine (CQ, 10μmol/L) treatment for 24 hours. Shown are 2 representative blots from the samples of 6 patients. The bar graph represents the relative PARP cleavage/GAPDH ratio measured by immunoblotting. (**B**, **C**) Flow cytometry using Annexin V–FITC/PI staining for cell apoptosis analysis. Primary CLL cells were incubated with 10μmol/L chloroquine for 24 hours while MEC-1 cell line were incubated with indicated concentrations of chidamide for 24 hours. Representative data shown are from 9 patients and three independent experiments of MEC-1. The bar graphs showed the percentage of apoptotic cells. (**D**) CCK8 assay for detecting metabolically active cells. MEC-1 cell lines was incubated with indicated concentrations of chloroquine for 24 and 48 hours. Viability of cells compared with the corresponding controls was shown from three independent experiments. (**E**, **F**) MEC-1 cells were transfected with ATG5 or nontargeting scrambled siRNA as indicated. Expression levels of targeted gene were analyzed by Immunoblotting. Percentage of apoptotic cells was assessed by flow cytometry following Annexin V-FITC/PI staining.

If autophagy inhibition is a critical step in mediating the cytostatic and cytotoxic effects on CLL cells by chidamide, then other autophagy inhibitors should be able to reproduce this effect. To test this hypothesis, we first performed pharmacological inhibition of autophagy using chloroquine. Previous data have shown it inhibits autophagy by disturbing the degradation of autophagic substrates, leading to the accumulation of LC3-II and SQSTM1 (Figure 1A, 1D, 1E). Furthermore, we found that chloroquine induced apoptosis in primary CLL cells and MEC-1 cell line (Figure 4A–4C) and inhibited cell growth of MEC-1 in a dose-dependent manner (Figure 4D), suggesting that basal autophagy may be a survival mechanism in primary CLL cells. To confirm this finding, we performed siRNA-mediated knockdown of key autophagy gene *ATG5* according to published researches conducted in CLL [22, 23]. In the MEC-1 cell line, despite the limited transfection efficiency, the introduction of siATG5 resulted in a certain degree of reduced target gene expression and reduced cell viability (Figure 4E, 4F). Therefore, autophagy may play a protective role in CLL cells, thereby providing a potential target for chidamide to play an anti-tumor effect on CLL cells.

### Chidamide and ibrutinib have synergistic effect on CLL cells

**Figure 5:**
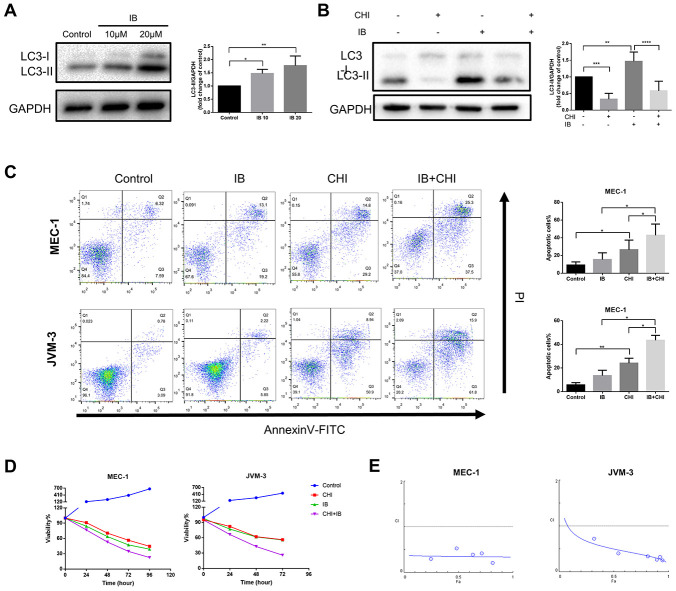
**Chidamide and Ibrutinib have synergistic effect on CLL cells.** (**A**) Immunoblotting analysis of LC3 in MEC-1 cell line cells after ibrutinib (IB, indicated concentrations) treatment for 48 hours. Representative data shown are from three independent experiments. The bar graph represents the relative PARP cleavage/GAPDH ratio measured by immunoblotting. (**B**) CCK8 assay for detecting metabolically active cells. MEC-1 and JVM-3 cell lines were incubated with indicated regimens (10μmol/L Ibrutinib, 20μmol/L chidamide alone or in combination) for 24 hours. Viability of cells compared with the corresponding controls was shown from three independent experiments. (**C**) Flow cytometry using Annexin V–FITC/PI staining for cell death analysis as mentioned before. MEC-1 and JVM-3 cell lines were incubated with indicated regimens (10μmol/L Ibrutinib, 20μmol/L chidamide alone or in combination) for 24 hours. The bar graphs showed the percentage of apoptotic cells. (**D**) Combination analyses were performed following the median-effect method 37. MEC-1 and JVM-3 cell lines were exposed to chidamide and Ibrutinib whose concentration were in a constant ratio of 2:1 simultaneously for 24 hours. The concentrations of chidamide used for these experiments were (in μmol/l) 1/2/10/20/40/50 while those of ibrutinib were (in μmol/l) 0.5/1/5/10/20/25 (shown as blue dots, and increasing from left to right along the x axis). CIs for different levels of growth inhibition (fraction affected) were calculated using the CompuSyn software. Details of CI and DRI values shown in Table 1. (**E**) Immunoblotting analysis of LC3 in MEC-1 cell line cells after ibrutinib (IB, 10μmol/L) treatment for 24 hours in the presence or absence of chidamide (CHI, 20μmol/L). The bar graph represents the relative expression level of LC3-II with respect to the control group.

Studies in glioblastoma and skin cancer found that Bruton’s tyrosine kinase (BTK) inhibitor ibrutinib induced protective autophagy, and inhibition of autophagy could increase the efficacy of ibrutinib [24, 25]. A recent study also showed that the expression of LC3-II increased in CLL patients during treatment with ibrutinib [26]. Consistent with the above studies, we also observed that ibrutinib increased the expression of LC3-II in MEC-1 cell line (Figure 5A). Therefore, we hypothesized that chidamide may enhance the efficiency of ibrutinib by inhibiting autophagy.

**Table 1:** Combination and does-reduction index of chidamide and ibrutinib.

**Fa**		**MEC-1**				**JVM-3**		
		**CI value**	**DRI IB**	**DRI CHI**		**CI value**	**DRI IB**	**DRI CHI**
0.5		0.35	4.44	7.98		0.50	4.11	3.91
0.75		0.35	4.75	7.43		0.38	6.37	4.50
0.9		0.34	5.09	6.93		0.29	9.87	5.17
0.95		0.34	5.33	6.61		0.25	13.29	5.69
0.97		0.34	5.51	6.40		0.23	16.43	6.09

Fa: Fraction Affected

CI: Combination Index; additive effect (CI=1), synergism (CI<1), and antagonism (CI>1)

DRI: dose-reduction index

IB: Ibrutinib

CHI: Chidamide

To test this hypothesis, we treated CLL cells with a combination of chidamide and ibrutinib. Such therapy further induced both apoptosis and growth inhibition (Figure 5C, 5D). Combination analyses were performed following the median-effect method [27]. The combination index (CI) calculated using the CompuSyn software confirmed the synergistic effect (CI<1) in this combinatorial therapy quantitatively (Figure 5E, Table 1). The results also showed favorable dose-reduction (DRI>1), indicating the potential to reduce toxicity by decreasing the doses of both drugs (Table 1). We further examined the effect of treatment on autophagy. Chidamide abrogated increased expression of LC3-II induced by ibrutinib (Figure 5A). These data demonstrate that chidamide-mediated autophagy inhibition may contribute to enhance the potency of ibrutinib.

## DISCUSSION

Various molecular drugs have led us into the novel agent era of CLL. However, there are remaining and newly raised problems need to work on. For elderly patients, especially those with comorbidities, who are unable to tolerate chemotherapy and long-term continuous treatment, the future direction may be the combination of targeted new drugs for fixed treatment time. For those younger patients, the main goal of treatment may be deeper remission together with reduced long-term side effects. In addition, efforts should be made to study the molecular mechanisms of relapse after treatment of new targeted drugs and to explore effective treatment. In recent years, histone deacetylase inhibitor (HDACi) has attracted more and more attention as a low-toxic and effective anti-tumor drug. Members of the HDAC family are associated with one or more cancer entities via diverse roles in pivotal cellular processes, including modulation of autophagy, both induction and inhibition of autophagic flux [28]. Several studies have shown abnormal levels of autophagy and its role in response and resistance to treatment in leukemias including CLL [13–16, 22].

We have investigated the effect of chidamide on autophagy in CLL cells and observed a significant decrease in the number of autophagosomes. By using chloroquine as a "tool" to impair lysosomal acidification, our analysis revealed that such a decrease is due to inhibition of autophagic flux. The consistent effect on cell lines and primary CLL cells is suggestive of a special role of chidamide for autophagy modulation. Recent studies in vitro have raised the hypothesis that HDAC family members are important for the regulation of autophagy at multiple levels. The HDAC family is divided into four subgroups. In most studies, depletion of class I and class IIa HDAC isozymes was associated with increased expression of autophagy regulators [29–31], whereas inhibition of class IIb HDACs was more likely to inhibit autophagy flux [32–35]. Interestingly, simultaneous deletion of HDAC1 and HDAC2 in mice blocked the induction and formation of autophagosomes [36]. Moreover, in the studies using pan-HDACi, autophagy was induced in various cancer cells [37, 38]. Exceptions were studies of hematologic malignancies, pan-HDACi inhibited autophagy in myeloid leukemia [9]. As for CLL, El-Kuli et al. reported that Class I HDACi (MGCD0103) inhibited autophagy, leading to apoptosis in CLL cells [22]. These results point to complicated prediction and interpretation due to different roles of HDAC isozymes and various cell types. Our findings now add new evidence that chidamide, a Class I and IIb (HDAC 1, 2, 3, 10) inhibitor, inhibited autophagy in the context of CLL cells.

Just as the role of autophagy in cancer is complex, so does the modulation of autophagy by different members of the HDAC family. They not only regulate the essential genes of autophagy through the deacetylation of histone or cytoplasmic proteins [39]. Besides, they can also regulate autophagy indirectly via the stress-induced response or modulation of the rapamycin (mTOR) pathway. In CLL, El-Khoury et al reported that type I HDACi (MGCD0103) can degrade autophagy-associated proteins by activation of calcium-activated neutral protease (calpain) [22]. Our analysis revealed for the first time that chidamide regulates autophagy in CLL cells independent of the PI3K/AKT/mTOR pathway. And instead of transcriptional regulation, such effect results from the interference with synthesis of autophagy-related proteins.

In terms of treatment potential of chidamide on CLL, there showed both cytostatic and cytotoxic effects. When combined chidamide with ibrutinib, we observed significant synergism with CI<1 and DRI>1, which also pointed to the potential of dose reduction to reduce toxicity. To propose a rational combination therapy, it is necessary to depict the molecular basis behind the effect. We and others speculated that ibrutinib induced autophagy in tumor cells including CLL cells [24–26]. And autophagy seems to help protect tumor cells from death and treatment-induced stress by the evidence that inhibition of autophagy promoted antitumor activity [24, 25]. We also found that the level of autophagy increased in primary CLL cells [13], and inhibition of autophagy recapitulates the effect of chidamide, again pointing to the protective role of autophagy in CLL cells. The current study also showed chidamide inhibited ibrutinib-induced autophagy, suggesting that chidamide may sensitize the CLL cells to ibrutinib by modulating autophagy.

Our research still has some limitations, such as the current validation of the effects of chidamide on autophagy protein synthesis and synergism of chidamide and ibrutinib were conducted in cell lines, which may not accurately represent the complex in vivo changes to the tumor. Although the *in vivo* model of CLL is difficult to establish accurately, further analyses using a co-culture system may offer more information. In addition, the influence of ibrutinib on autophagy and the change of autophagic flux after the combination treatment of ibrutinib and chidamide need further verification besides the expression level of LC3-II. Further studies on the mechanism behind are also needed to elucidate the role of autophagy in the treatment of CLL, and the potential role in resistance to ibrutinib, which may be helpful to identify patients most likely to benefit from such combination therapy. Analyses of more autophagy-related (ATG) genes and Atg-related proteins which are essential for the formation of canonical autophagosomes, and exploration of factors which modulate the translation rate including the binding of proteins to regulatory elements on the transcript or the binding of non-coding RNAs such as micro-RNAs may be the directions for the further study.

The evidence presented in this paper indicates that in CLL cells, the increase of autophagy may be one of the mechanisms of the enhanced survival and apoptosis of CLL cells. Chidamide has cytostatic and cytotoxic effects on CLL cells at least partially by regulating autophagy activity at the post-transcriptional level. Furthermore, the combination of chidamide and ibrutinib shows a significant synergistic effect together with a decrease of ibrutinib-induced autophagy.

## MATERIALS AND METHODS

### Ethics statement

The experiments were following with the ethical standards of the Declaration of Helsinki. This study was approved by Institutional Review Boards of the First Affiliated Hospital of Nanjing Medical University. Primary CLL cells were obtained from the peripheral blood of patients after written informed consent.

### Patient sample and cell lines

A total of 25 newly diagnosed CLL patients were enrolled from January 2017 to March 2018. The clinical features of patients were summarized in Supplementary Table 1. The inclusion criteria included: (1) B-CLL confirmed according to guidelines updated in 2008 by International Workshop on Chronic Lymphocytic Leukemia [40]; (2) Age 18-70 years inclusive; (3) Written informed consent. While the exclusion criteria included: (1) Any previous treatment for CLL; (2) Richter’s transformation. Peripheral blood mononuclear cells were separated by density gradient method and lymphocyte separation liquid was from Jing Yang Company (Tianjin, China). CD19 positive cells were sorted using the Magnetic Activated Cell Sorting method (MACS) with CD19 magnetic beads from Miltenyi Company (Bergisch Gladbach, Germany). The MEC-1 and JVM-3 were obtained from the American Type Culture Collection. PBMCs and cell lines were cultured in Iscove’s Modified Dulbecco’s medium (HyClone, Logan, UT, USA), containing 10% fetal bovine serum. The cells were incubated at 37 °C in an atmosphere of 5% CO2. Cells from the same culture flask were used for experiments without starvation before drug treatment.

### Drugs and inhibitors

Chidamide was provided by Chipscreen Biosciences (Shenzhen, China). Ibrutinib and MG132 were purchased from Selleck Chemicals (Houston, TX, USA). Chloroquine (CQ) and cycloheximide (CHX) were purchased from Sigma-Aldrich (St. Luis, MO, USA).

### Western blotting and antibodies

Cells were lysed in RIPA buffer (Beyotime, Shanghai, China) supplemented with protease and phosphatase inhibitor cocktails (Bimake, Houston, TX, USA). Proteins extracted from whole-cell lysates were resolved by SDS polyacrylamide gel electrophoresis and transferred to nitrocellulose membranes. Antibodies used for immunoblots included LC3A/B, PARP, mTOR, Phospho-mTOR (Cell Signaling Technology, Danvers, MA, USA), p62 (SQSTM1, Abnova, Taoyuan, China), GAPDH (Proteintech, Chicago, IL, USA). Species-appropriate secondary antibodies were obtained from Jackson ImmnuoResearch (Philadelphia, PA, USA). After antibody incubations, proteins were detected using a chemiluminescent substrate (Millipore, Darmstadt, Germany).

### RNA extraction and quantitative RT–PCR

Total cellular RNA was extracted and synthesized to cDNA. Pubmed genebank showed the mRNA sequences, and then we designed the primers (details were shown in Supplementary Table 2) and used GAPDH as a reference gene (Primers were synthesized by the United States Invitrogen Corporation, Shanghai branch). The expression level of indicated mRNA was presented as relative fold change (2^−ΔΔCt^) with the control group normalized to a fold value of 1. ΔCt was used to measure statistical significance.

### Transmission electron microscopy

Cells were fixed with Fixative for TEM (Servicebio, Wuhan, China). Samples were post-fixed using 1% OsO4 in 0.1 M PBS (pH 7.4), followed by dehydration with an increasing concentration gradient of ethanol and acetone. Then, samples were infiltrated, embed, cut into 60−80 nm sections, and stained with uranyl acetate and lead citrate. Images were acquired using a transmission electron microscope (HITACHI, HT7700).

### Cell growth inhibition assay

Cells were seeded in 96-well cell culture plates and assays were performed in triplicate. Cell viability was measured using the Cell Counting Kit-8 (CCK-8) assay Kit (Dojindo, Kumamoto, Japan). Absorbance from each well was read and used to calculate the percentage of viability following the equation below, then the growth curve was drawn. $Viability\%=\frac{A_{sample}-A_{b}}{A_{c}-A_{b}}\times 100\%$ (A = absorbance, Blank: Ab, Negative control: Ac). Drug combination analysis was performed following the median-effect method [27] using CompuSyn software. Combination index (CI) <1, CI=1, and CI >1 indicate synergism, additive effect, and antagonism, respectively. Cell viability was calculated as 100% × (absorbance of the treated wells – absorbance of the blank control wells) / (absorbance of the negative control wells – absorbance of the blank control wells) (A represents the absorbance recorded at 450nm). Half maximum inhibitory concentration (IC50) values are presented as mean ± standard deviation (SD).

### Cell apoptosis assay

An annexin V-FITC /propidium iodide double stain assay (BD Biosciences, San Jose, CA, USA) by flow cytometry was performed to detect apoptosis induction following the manufacturer’s protocol. Seeded cells were incubated with single-agent or combination therapy in different combinations as indicated. Data were analyzed with FlowJo 10.

### siRNA transfections

Knockdown of ATG5 was performed by transfection of MEC-1 cells with small interference RNAs (siRNAs). The siRNA sequences were shown in Supplementary Table 3. Lipofectamine 3000 reagent (Invitrogen, Carlsbad, CA) and siRNA oligo were diluted in Opti-MEM Medium and mixed well. Then the mixture was added to seeded cells for transfection.

### Statistical analysis

Statistical analysis was carried out using GraphPad Prism. All data were tested for normal distribution and variance homogeneity. Differences between 2 groups were compared using a two-tailed, unpaired Student’s t-test. Mann-Whitney was applied to analyze data with a skewed distribution. One-sample t-test was used to compare the mean of one sample to a standard control while the Wilcoxon rank sum test was used for a skewed distribution. One-way ANOVA was used for comparisons among multiple groups, whereas data with a skewed distribution were examined with a Kruskal-Wallis H test. A value of P<0.05 indicated statistical significance.

## Supplementary Material

Supplementary Tables
