# Supplementary material for: Chidamide, a histone deacetylase inhibitor, inhibits autophagy and exhibits therapeutic implication in chronic lymphocytic leukemia
Source: Aging (Albany NY). 2020 Aug 27;12(16):16083–98. doi: 10.18632/aging.103536 (PMC7485718; doi:10.18632/aging.103536)
Supplement: Supplementary Tables [file aging-12-103536-s001..pdf]

SUPPLEMENTARY TABLES

Supplementary Table 1. Clinical characteristics of the CLL patients.

Patient n	Age (y)	Sex	Rai stage	Binet Stage	ZAP-70 status	CD38 status	IgV_H status	TP53 mutation	Cytogenetics
1	51	F	1	A	NA	NA	unmutanted	unmutanted	del13q
2	49	M	0	A	NA	NA	mutated	unmutanted	t(14;18)(q32;q21)
3	49	M	4	C	-	-	unmutanted	unmutanted	complex chromosomal abnormalities
4	57	M	1	A	+	-	NA	NA	Normal
5	52	M	1	A	+	-	unmutanted	NA	del13q
6	86	M	1	A	-	-	unmutanted	unmutanted	Normal
7	56	M	1	A	-	NA	unmutanted	unmutanted	del11q, del13q
8	62	M	4	C	+	NA	unmutanted	mutated	del13q
9	66	M	1	A	-	-	unmutanted	unmutanted	complex chromosomal abnormalities
10	53	M	2	B	+	-	NA	unmutanted	Normal
11	64	M	3	C	+	+	unmutanted	NA	del13q
12	61	M	3	C	+	NA	mutated	NA	Normal
13	62	F	2	B	NA	NA	NA	1	complex chromosomal abnormalities
14	47	M	0	B	-	+	mutated	unmutanted	complex chromosomal abnormalities
15	59	F	4	C	-	-	unmutanted	unmutanted	del13q
16	65	M	2	B	-	-	NA	1	+12
17	71	M	2	B	NA	-	unmutanted	1	del13q, del17p
18	31	M	1	A	+	+	mutated	unmutanted	del11q
19	65	M	4	C	-	NA	NA	unmutanted	+12
20	59	M	1	B	NA	-	unmutanted	unmutanted	Normal
21	64	M	0	A	+	+	unmutanted	unmutanted	del13q
22	52	M	1	A	+	-	unmutanted	NA	del13q
23	25	F	4	C	+	+	NA	unmutanted	complex chromosomal abnormalities
24	37	M	2	B	+	+	mutated	NA	Normal
25	67	F	3	B	+	NA	unmutanted	unmutanted	del13q

NA indicates information not available.

Supplementary Table 2. Primers for quantitative RT PCR.

Gene	Primer
LC3	Forward -5'- AACATGAGCGAGTTGGTCAAG -3'
	Reverse-5'- GCTCGTAGATGTCCGCGAT-3'
SQSTM1	Forward-5'- GACTACGACTTGTGTAGCGTC-3'
	Reverse-5'-AGTGTCCGTGTTTCACCTTCC-3'
ATG7	Forward-5'- ATGATCCCTGTAACTTAGCCCA-3'
	Reverse-5'-CACGGAAGCAAACAACTTCAAC-3'
ATG3	Forward-5'- ACATGGCAATGGGCTACAGG-3'
	Reverse-5'-CTGTTTGCACCGCTTATAGCA-3'
GAPDH	Forward-5'-TGGGTGGAATCATATTGGAAC-3'
	Reverse-5'-TCAACGGATTTGGTCGTATTG-3'

Supplementary Table 3. siRNA sequence.

	Primer
si-ATG5-homo-938	Sense -5'- GACCUUUCAUUCAGAAGCUTT -3'
	Antisense-5'- AGCUUCUGAAUGAAAGGUCTT-3'
si-ATG5-homo-695	Sense -5'- GUCCAUCUAAGGAUGCAAUTT-3'
	Antisense -5'-AUUGCAUCCUUAGAUGGACTT-3'
si-ATG5-homo-486	Sense -5'- GACGUUGGUAACUGACAAATT-3'
	Antisense -5'-UUUGUCAGUUACCAACGUCTT-3'
si-GAPDH	Sense -5'- UGACCUCAACUACAUGGUUTT-3'
	Antisense -5'-AACCAUGUAGUUGAGGUCATT-3'
Negative control	Sense -5'-UUCUCCGAACGUGUCACGUTT-3'
	Antisense -5'-ACGUGACACGUUCGGAGAATT-3'
